# Advances in Stem Cell Research—Insights from the Special Issue “Stem Cells in Health and Disease 2.0”

**DOI:** 10.3390/ijms252413364

**Published:** 2024-12-13

**Authors:** Anne-Marie Galow, Francesca Agriesti

**Affiliations:** 1 Research Institute for Farm Animal Biology (FBN), 18196 Dummerstorf, Germany; 2 Department of Clinical and Experimental Medicine, University of Foggia, 71122 Foggia, Italy

Significant progress in stem cell research over the last few decades has opened up new avenues for exploring human physiology and pathophysiology. In fact, the importance of stem cells in both basic research and therapeutic applications is indisputable. Broadly, stem cells can be categorized into two main types: embryonic stem cells (ESCs), which are derived from blastocysts and can differentiate into any cell lineage, and adult stem cells, which are resident in various tissues but have more restricted differentiation potential.

Embryonic stem cells have several advantages, including pluripotency, higher proliferation rates, stable morphology, and telomerase activity during long-term cultivation. However, their clinical use is limited due to ethical concerns and their reported tumorigenic potential [1]. In 2006, a groundbreaking alternative emerged with the development of induced pluripotent stem cells (iPSCs) by Yamanaka et al. [2]. Through the introduction of the four transcription factors *Oct4*, *Sox2*, *Klf4*, and *Myc*, somatic cells can be reprogrammed into embryonic-like pluripotent stem cells that are capable of differentiating into any cell type from all three germ layers. This innovation has propelled advancements in disease modeling and clinical research.

On the other hand, adult mesenchymal stem cells (MSCs) have demonstrated remarkable versatility in therapeutic applications. MSCs, a heterogeneous group of stromal cells, exhibit self-renewal and multilineage differentiation potential [3]. These cells can be easily isolated from various sources, such as bone marrow, adipose tissue, umbilical cord blood, and peripheral blood, making them valuable in stem cell-based therapies.

This Special Issue, entitled “Stem Cells in Health and Disease 2.0”, features thirteen papers that provide in-depth insights into the current focal points of stem cell research and transformative applications in regenerative medicine, disease modeling, and therapeutic interventions. Currently, the two main fields of stem cell applications are in vivo cell therapy via cell replacement and immunomodulation and in vitro disease modeling and drug testing. The excellent reviews conducted by Bruno et al. and Bakinowska et al. summarize the role of gestational stem cells in the treatment of neurological disorders and the applications of MSCs and iPSCs in addressing cardiovascular disease, respectively.

The immunomodulatory properties of MSCs significantly contribute to the success of stem cell-based therapies. Accordingly, Fang et al. demonstrated that combining an IL-6 receptor-blocking antibody with bone marrow-derived MSCs reduced mature B cell proportions while increasing the number of Treg cells, thereby attenuating humoral immune responses in an allo-sensitized mouse model. This finding provides further evidence for the pleiotropic applications of MSCs.

To reflect the recent advancements made in this field, the International Society for Stem Cell Research recently updated its Guidelines for Stem Cell Research and Clinical Translation [4]. However, challenges such as standardizing definitions, protocols, and reliable markers persist. To address these gaps, Engelbrecht et al. developed a convolutional neural network to distinguish between leukemic and normal hematopoietic stem cells. Their study achieved a 93.42% accuracy rate using brightfield and DNA imaging, thereby showcasing the potential of artificial intelligence in advancing stem cell diagnostics. Notably, the resulting performance metrics varied between patients.

Despite interindividual variability complicating statistical analyses, patient-derived stem cells have become indispensable in disease modeling and drug discovery. For instance, Hussein et al. characterized MSCs derived from the osteoarthritic knee joints of diabetic patients, evaluating the impact of comorbidities on clonogenicity, proliferation, and surface marker expression. While their immunophenotype and proliferative potential were similar, a reduced response to osteogenic induction was observed. In another study, Claeys et al. generated MSCs via an intermediate neural crest cell stage using iPSCs from Osteogenesis Imperfecta patients. These MSC-derived osteoblast-like cells displayed reduced collagen expression and mineralization, mirroring the disease pathology. Damiani et al. and Gois Beghini et al. have also provided further insights into the applications of iPSCs in neurodegenerative disease modeling and drug discovery.

Beyond traditional MSCs and iPSCs, alternative sources of stem cells are gaining attention. Menstrual blood-derived endometrial stem cells, for example, exhibit significant therapeutic potential, as outlined by Cordeiro et al. These authors explore how menstrual blood-derived stem cells can be applied in vaginal atrophy therapy among BRCA1/2 cancer-risk patients that have undergone a risk-reducing bilateral salpingo-oophorectomy. Additionally, they discuss the therapeutic potential of exosomes derived from menstrual blood stem cells to counteract premature ovarian insufficiency induced by systemic anticancer treatments.

Stem cell-derived exosomes have garnered significant interest due to their advantages, including their non-immunogenicity, low infusion toxicity, easy accessibility, and lack of tumorigenic risks [5]. In this Special Issue, Jang et al. demonstrated the beneficial effects of MSC-derived extracellular vesicles in stroke models, showing improved neuronal viability, reduced cerebral infarction size, and better forelimb usage in Sprague–Dawley rats. Interestingly, MSCs treated with blueberry extract produced more extracellular vesicles, which resulted in more elongated neurofilaments and neurons compared to the untreated MSCs and controls, implying improved neuroprotection.

Even though stem cells have proven to be reliable in achieving therapeutic effects, there are still gaps in our mechanistic understanding of their actions and features. Lin et al. identified Dachshund homolog 1 as having a key role in suppressing the anthrax-induced inhibition of megakaryocytic differentiation in hematopoietic stem cells. Interestingly, the differentiation of stem cells is interlinked with the circadian clock. As highlighted in a study by Agriesti et al., PSCs lack a discernible circadian clock rhythm, a feature they acquire during differentiation, due to complex regulatory mechanisms such as post-transcriptional repression and epigenetic modifications. This fascinating phenomenon offers insights into how temporal cues influence stem cell identity and the delicate balance between pluripotency, differentiation, and regenerative capacities, opening up new avenues in stem cell-based therapies.

While the therapeutic applications of stem cells are promising, significant challenges remain. Standardizing protocols, exploring minimally invasive cell sources, and deepening our understanding of molecular mechanisms will be key to translating this research into clinical realities. This Special Issue not only highlights the current state of this field of research but also sets the stage for future innovations that could redefine modern medicine. By bridging fundamental research and clinical applications, the contributions in this Special Issue offer valuable insights into current stem cell research. Future studies should aim to deepen our understanding of stem cell mechanisms, explore alternative sources of stem cells that are accessible through minimally invasive methods, and standardize clinical protocols for tissue engineering and other therapeutic approaches.
